# Teeth Extracted without Pain

**Published:** 1867-06

**Authors:** 


### Editorial.

### "TEETH EXTRACTED WITHOUT PAIN."

In the Dental profession, as in the world, there are two kinds of people. In regard to the pain inflicted by cutting out sensitive dentine, extirpating the pulp, or driving a wedge between the necks of the teeth, I have heard heroic operators complacently remark, "the pain's the patient's business. Mine is to make a good operation." And this is true, but not to the unfeeling extent to which it is carried, by those with whom it is a favorite expression. The other class, through a too ardent desire to avoid the infliction of present pain, often disregard the patient's best interests; and of the two errors, this is by far the more serious.

In the present condition of the race, the amount of suffering directly resulting from morbid dentition, and diseased Dental organs, is so great, and its character so severe, that simply to think of it is appalling. The rueful countenance, the piteous wail, the convulsive shudder, the stupor, and the death of the little babe, are sad comments on the condition of fallen man. "Who did sin—this man or his parents, that he was born blind," is a melancholy query propounded to him who spake as never man spake. The light of science corroborates the teaching of inspiration that the iniquities of the fathers are visited upon the children.

It is, then, to be expected that for several generations yet to come, defective teeth will be found, and some so defective that their restoration to health will not be practicable. And as teeth diseased beyond a slight morbidity are absolutely intolerable, at least occasional extraction will be indicated, and will even be necessary, and if necessary—pain or no pain—"that's the question."

At present, the extraction of teeth is more frequently required than are all other surgical operations combined. And, however, lightly some may regard it, the extraction of a tooth is a severe operation. It is painful, and produces a decided nervous shock. The teeth are, mechanically considered, very firmly attached to the organism. Their vital connection is also very decided. I have known men who neither moaned nor murmured at being mangled by bullets, who, nevertheless, shrieked and trembled from the pain of ordinary tooth-extraction. In most cases, the extraction of a dozen teeth is as severe as the amputation of an arm, and the consequences are less serious, only on account of the difference in the structure and vitality of the parts mutilated. The Surgeon, who would, under ordinary circumstances, amputate an arm without resorting to anæsthesia, would be guilty of malpractice. Is the Dental Surgeon, then, to be criticised and condemned for a similar resort in an operation equally severe? Or, because his operation is divisible, must he reduce the vitality of his patient, by a series of a dozen painful operations, with as many nervous shocks, to say nothing of the mental anguish, protracted through the several intervals? Such Surgery is but little if at all better than that of the boy who cut off the dog's tail, an inch at a time, regarding the removal of the entire organ at once, as quite too severe.

But the quotation with which this is headed, is regarded by some as a baneful announcement to afflicted humanity. Even with pain, far too many teeth are extracted, say they; and no honest, well-informed man will dispute this. But if this is meant as an argument against painless extraction, it proves too much. The improved forceps must be thrown aside, and the barbarous (or *barber*-ous) turnkey resumed. Men of science and skill must cease to operate, and leave extraction to the blacksmith and the butcher. By a good deal of management, the operation might be made far more terrible than ever, and even then, it is probable teeth would be extracted that ought to be saved. If sound here, the argument is equally valid in general Surgery. Anæsthesia must be abandoned, for too much cutting is done. Patients insist on having organs removed that might be saved. It will not be safe or proper to mitigate the pains of maternity, for as matters stand even now, too many babes are born in some families.

As an admirer of human organism in all its integrity, I yield to no one. I never remove an organ, or mutilate the system in any way, without a feeling of sadness. If any one is more conscious than I, that thousands of teeth are daily sacrificed on the altars of avarice and ignorance, I will sit at his feet and listen to his recitals, as a child listens to the tale of midnight murder. But as long as any mutilation is necessary, let that be painless whenever practicable. I know it is urged by some that pain is a blessing. Certainly not a blessing *per se*—in its own essence. Though ignorant of what freedom from pain is, my faith takes hold of the promise, "There remaineth a *rest* to the people of God," not a *pain;* that is not reserved for them, as it would be if a blessing.

The removal of Adam's rib took place while he was in an anæsthetic state, and it is a wonder that Surgery has been so slow to take the hint legitimately deducible from this fact. It is to be hoped that the day is not far distant when all Surgical operations will be painless.

